# Evolution of the Food Web in Bandon Bay, the Gulf of Thailand: Ten Years of the Blue Swimming Crab Stocking Programme

**DOI:** 10.21315/tlsr2023.34.2.6

**Published:** 2023-07-21

**Authors:** Amonsak Sawusdee, Sontaya Koolkalya, Thanitha Thapanand, Tuantong Jutagate

**Affiliations:** 1 School of Engineering and Resource Management, Walailak University, Thasala, Nakhon Si Thammarat 80160, Thailand; 2 Faculty of Agricultural Technology, Rambhai Barni Rajabhat University, Mueang Chanthaburi District, Chanthaburi 22000, Thailand; 3 Faculty of Fisheries, Kasetsart University, Chatuchak, Bangkok 10900, Thailand; 4 Faculty of Agriculture, Ubon Ratchathani University, Warin Chamrap, Ubon Ratchathani 34190, Thailand

**Keywords:** Crab Fisheries, Blue Swimming Crab, Crab Bank, Ecopath, Biomass, Impact

## Abstract

The ecosystem of Bandon Bay, in the Gulf of Thailand (GoT), has been impacted since 2007 by the continued stocking of larval blue swimming crab *Portunus pelagicus*, also called a crab bank. In this study, the food web in the Bay was modelled using Ecopath software to compare the trophic status, interaction and energy flow among the components in the system in 2007 and 2016 (i.e., before and 10 years after the crab bank intervention). The models were based on data collected from trawling. Twenty fish and shellfish components were used in the 2007 model, while 22 were used in the 2016 model. A significant increase in biomass was found in blue swimming crab, but biomass declined for other demersal fishes, cephalopods, and Penaeid shrimps. The production/biomass ratios of most components were higher in 2016 but the consumption/biomass ratios were relatively unchanged. The ecotrophic efficiency indicated that shellfishes were more exploited than fishes. Changes in most of the ecological indices revealed higher maturity and stability after 10 years of crab bank operation. The mixed trophic impact indicated bottom-up regulation, and that the increase of blue swimming crab negatively impacted only Mantis shrimp. Overall, the results indicate positive impacts of the crab bank intervention.

**Highlights**

- Significant increase in biomass of the blue swimming crab after 10 years of stocking programme in Bandon Bay, the Gulf of Thailand.
- The results of Ecopath model revealed higher maturity and stability after 10 years of blue swimming crab stocking programme.
- The mixed trophic impact indicated bottom-up regulation, and that the increase of blue swimming crab negatively impacted only Mantis shrimp.

## INTRODUCTION

The Gulf of Thailand (GoT) is among the most productive large marine ecosystems; the marine capture fisheries within Thai territory of the GoT contribute over 65% of the country’s total marine production (about 2.5 × 10^6^ tonnes) each year (Lymer *et al*. 2008). The fisheries in the GoT are intensive, both in inshore and offshore areas. Thus, declines in biomass of many fisheries-targeted species are observed, which necessitate appropriate fisheries management that can balance both economics and environmental paradigms (Koolkalya *et al*. 2015; Satumanatpan & Pollnac 2017). Within the GoT, Bandon Bay, which is located in Surat Thani Province of southern Thailand, is one of the most important coastal areas for human activities, including fisheries. The bay has a coastline of 156 km with huge intertidal mudflats extending 2 km offshore, and it receives nutrients from numerous river channels. These factors make Bandon Bay an ideal habitat and fishing ground for many fishes and shellfishes, including the blue swimming crab *Portunus pelagicus*, which significantly supports the crab-meat industry in Thailand (Jarernpornnipat *et al*. 2003; Sawusdee 2010).

Similar to other fishery resources in the GoT, *P. pelagicus* has been heavily exploited due to the high demand of crab meat. The annual catch of this species is presently around 25,000 tonnes a year, but catches were as high as 40,000 tonnes in the 1990s (Kunsook *et al*. 2014). Due to the decline of the resource, a crab bank programme has been introduced. It is a kind of stocking programme in which gravid females are placed in onshore storage to release eggs, and then the larvae are reared before being released to the sea. The stage of release varies from site to site, ranging from zoea (1–2 days) to 20 days after hatching (Thiammueang *et al*. 2012; Nitiratsuwan *et al*. 2014). Enhancing fishery resources through release of cultivated species is considered one of the effective mitigations available in fisheries management (Ak *et al*. 2016), and the crab bank in the GoT appears to be a successful example. Since the introduction of the crab bank in early 2000 along both the GoT and Andaman Sea, several studies have shown a significant increase in abundance and catch rate of *P. pelagicus* at locations where this programme was implemented (Thiammueang *et al*. 2012; Arkonrat *et al*. 2013; Nitiratsuwan *et al*. 2014).

For Bandon Bay, most people (about 70%) who live along the coastal area of the bay are involved in either fisheries or mariculture industries. Catch composition from combined fishing activities, mostly at an artisanal level, showed that catches could be as high as 50%, followed by squids, pelagic fishes, demersal fishes and crabs (Sawusdee 2010). In terms of crabs, more than 85% of the yield is *P. pelagicus*, which are caught by two main fishing gears, namely collapsible traps and bottom-set gillnets (Jutagate & Sawusdee 2022). The catch per unit effort of *P. pelagicus* in Bandon Bay showed a drastic decline in early 2000, i.e., from more than 1 kg/h to less than 0.1 kg/h. At the same time, the average carapace width of the harvested crabs became smaller, i.e., less than 10 cm compared to about 12 cm in the 1990s (Sawusdee 2010; Niumnuch & Purisumpun 2011). Due to the decline of the resource and based on the success of a crab bank project demonstrated in Chumporn Province in the early 2000s (Thiammueang *et al*. 2012), this stocking programme was applied by the Department of Fisheries to Bandon Bay starting in 2007. Presently, crab banks are operated in Bandon Bay not only by Department of Fisheries (DoF), but also other sectors including private companies, provincial and district organisations, non-governmental organisations (NGOs), and even fishing communities.

One of the most serious concerns for a stocking programme, including the crab bank, is whether this activity causes changes in the abundance of other species in the system, which could consequently lead to imbalance of populations and possibly result in the loss of other ecosystem values and services (Caddy & Defeo 2003; Molony *et al*. 2003; Bell *et al*. 2006). This imbalance is mainly from two causes: competition and predation. Competition for food resources occurs both at the intraspecific level, due to increased abundance of individuals by the addition of hatchery-reared seeds, and at the interspecific level, due to competition between hatchery reared seeds and other species with similar ecological requirements and potentially leading to a reduction in abundance of competing species and prey species (Molony *et al*. 2003). Predation can occur either by or to the stocked species, which may result in trophic cascades, or community-level cascades (Polis *et al*. 2000) that impact at least three trophic levels and can extend to any multilink linear food-web interaction (Caddy & Defeo 2003). Moreover, exceeding the carrying capacity of the ecosystem due to continued stocking is also considered a cause of imbalance (Blaxter 2000; Molony *et al*. 2003). Therefore, quantification of the impacts of stocking programmes, such as the crab bank, on the ecosystem is an important step in determining the appropriateness of particular management actions (Fayram *et al*. 2006; Khan *et al*. 2015).

Understanding of food web structure and ecosystem dynamics is important for determining the interactions in an ecosystem and useful to many ecological studies (Khan *et al*. 2015). Several mass-balance models have been applied for the purpose of understanding ecosystem processes and how they govern the living components in the system. Among the mass-balance models, Ecopath (Polovina 1984) is the most popular and widely applied for estimating the biomass budget for each component in the ecosystem, together with their mortality, diet and energetics parameters. Ecopath partitions the ecosystem into boxes representing a component, i.e., a species or a group of species that have similar life history. The software analyses interactions among components as well as provides quantitative descriptions of the structure of food webs of the system. In doing so, Ecopath works under the assumption that the ecosystem under consideration is at equilibrium, i.e., inputs to a component should equal outputs for the period being considered (Polovina 1984; Christensen *et al*. 2005). As a software for balancing steady-state model, it allows the user to make a comparative study between two periods of interest, in particular before and after intervention by human activities such as regulation measures; fisheries actions, damming, species introduction as well as stocking programmes (Christensen *et al*. 2005; Fayram *et al*. 2006; Khan *et al*. 2015).

This study, therefore, aims to describe two different situations of the Bandon Bay ecosystem in 2007 and 2016, and investigate the evolution of the ecosystem through its food web structure and ecosystem functioning in response to the stocking of *P. pelagicus* through the crab bank programme. It is worth noting that the year 2007 was the first year of the “crab bank” campaign in Surat Thani Province; later these crab banks were implemented more intensively along the coast of Bandon Bay (Sawusdee 2010). The study was done using the Ecopath with Ecosim (EwE) software version 6.2 (freely available at http://www.Ecopath.org; Christensen *et al*. 2005). The results can be further applied for policy development on the sustainable use of resources in Bandon Bay or for deriving management strategies for blue swimming crab fishing grounds elsewhere.

## MATERIALS AND METHODS

### The Study Area

**Figure 1:**
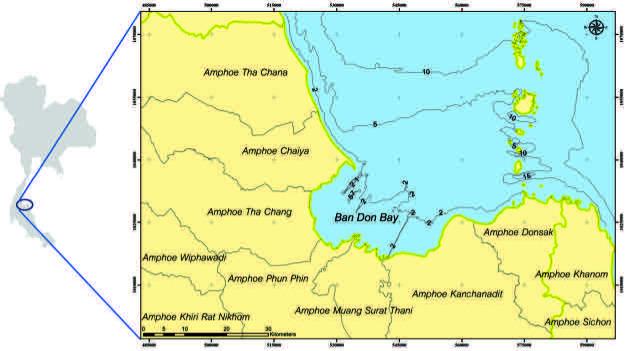
Location of the Bandon Bay.

Bandon Bay (9°12’ N; 99°40’ E), located in southern Thailand (Fig. 1), is the largest estuarine (*ca* 1,070 km^2^) and mangrove inlet on the east coast of Thailand, and empties into the GoT. This bay serves as a crucial nursery and feeding ground of many brackish and marine species and is considered a textbook example of excessive utilisation of coastal resources (Jarernpornnipat *et al*. 2003). Surface water currents in the bay show two significantly different patterns, according to season: counterclockwise circular patterns during the dry season, from January to March; and flowing southwards during the rainy season, from April to December (Wattayakorn *et al*. 1999). The coastal area is gradually sloped, and the average water level in the bay is 2.9 m, fluctuating from less than 1 m to 5 m (Wattayakorn *et al*. 1999; Jarernpornnipat *et al*. 2003).

### The Ecopath Model

Since the first introduction of the Ecopath model in 1984 in French Frigate Shoals (Polovina 1984), this model has been widely used to describe the trophic interactions and mass balance in aquatic ecosystems. It uses the Ecopath with Ecosim (EwE) software, and the model has been progressively improved, both in terms of software and techniques, by the University of British Columbia’s Fishery Centre (Christensen *et al*. 2008; Heymans *et al*. 2016). Details of Ecopath and instructions for constructing models with it can be obtained from the website, http://www.Ecopath.org, or by viewing examples of over 400 models published in various scientific journals (Colléter *et al*. 2015; Heymans *et al*. 2016). In brief, for the Ecopath model, it is assumed that the ecosystem is in steady-state for each component, i.e., inputs equal outputs, and the basic mass-balance concept (Christensen *et al*. 2005) can be described as:


(1) Production=catches+predation mortality+biomass accumulation+net migration+other mortalities

or written as a linear equation as:


(2) $$P_{i}=Y_{i}+B_{i}+M2_{i}+E_{i}+{BA}_{i}+P_{i}\times (1-{EE}_{i})$$

where, for any component (i), *P_i_* is the total production rate; *Y_i_* is the total fishery catch rate; *M2_i_* is the total predation rate; *B_i_* is the biomass; *E_i_* is the net migration rate (i.e., emigration – immigration); *BA_i_* is the biomass accumulation rate; *M*0*_i_ = P_i_ × *(1 − *EE_i_*) is the other mortality rate, and *EE_i_* is the ecotrophic efficiency (i.e., the fraction of the production that is utilised within the ecosystem by predators or exported or removed by fishery).

To construct the ECOPATH, the model is expressed in terms of utilisation of production of each component in the ecosystem at an arbitrary time period, and Equation (2) can be re-expressed as:


(3) $$B_{i}\times {\left(P/B\right)}_{i}\times {EE}_{i}=\sum_{j=1}^{n}B_{j}\times {\left(Q/B\right)}_{i}\times {DC}_{ij}+{EX}_{i}$$

where *(P/B)_i_* is the production/biomass ratio; *B_j_* is the biomass of predator *j; (Q/B)_j_* is the relative food consumption of *j*; *DC_ij_* is the fraction of prey *i* in the diet of predator *j*; *EX_i_* is the export from the ecosystem, mostly through fisheries.

From Equation (3), four parameters, namely *B_i_*, *(P/B)_i_*, *EE_i_* and *(Q/B)_j_*, as well as diet composition of each component are required as inputs to construct the ECOPATH. At least 3 out of 4 parameters must be input to the model for each component, and then *n* linear equations are created for *n* components and solved for the remaining parameter (Christensen *et al*. 2005).

### Model Structure

#### Model components

Details of component in the Ecopath analysis of the Bandon Bay ecosystem are in Table 1. Finally, there were 20 fish and shellfish components (i.e., species/group of species) used for constructing the Ecopath model of Bandon Bay in 2007, and 22 components for the 2016 model (Tables 2 and 3). These components represent the catch composition from trawl surveys by the research vessel of the Chumphon Marine Fisheries Research and Development Centre within the Bandon Bay area, which were six survey-cruises in 2007 and 10 in 2016.

#### Model inputs

Input parameters for the basic estimation in the Ecopath model are shown in Tables 2 and 3 and the details of each parameter are as follows:

1. Biomass (*B_i_*): biomass of each fish and shellfish component was estimated from the trawl survey data in Bandon Bay, conducted by Chumphon Marine Fisheries Research and Development Center of Department of Fisheries, in 2007 and 2016 by using the swept area method (Sparre & Venema 1992) as:
(4) $$B=\left(\frac{\bar{CpUE}}{a\times X_{1}}\right)\times A$$
  where $\bar{CpUE}$ is the average catch per unit effort of each component; *a* is the area swept by the trawl per hour (0.09029 km^2^); *X_1_* is the proportion of fish in the path of the gear retained by the net (0.5) and A is the total area of Bandon Bay (480 km^2^).
2. Production/Biomass ratio *(P/B)*: The *P/B* ratio was estimated through use of the instantaneous rate of total mortality (*Z*, year ^−1^) as described by Allen (1971). During the surveys, catch of each species was sampled and lengths of individuals were measured. Thus *Z* was estimated by Beverton and Holt (1957) as:
(5) $$Z=\frac{K(L_{\infty }-\bar{L})}{\bar{L}-L^{\prime }}$$
  where *L_∞_* is the asymptotic length (cm), *K* is the curvature parameter of the von Bertalanffy’s growth function, *L̄* is the mean length in the population (cm), and *L’* represents the mean length at entry into the fishery (cm).
3. Relative food consumption *(Q/B)*: The *Q/B* ratio was estimated from the empirical relationship proposed by Palomares and Pauly (1989) as:
(6) $$log(\text{Q}/\text{B})=7.964-0.204log{\text{W}}_{\infty }-1.965{\text{T}}^{\prime }+0.083\text{A}+0.532\text{h}+0.398\text{d}$$
  where *W_∞_* is the asymptotic weight (g), *T’* is the mean temperature of Bandon Bay at 29°C (expressed by T’ = 1000/*K* (*K* =°C + 273.15), *A* is the aspect ratio (*A* = *H*_2_/*S*; *H* is the height of caudal fin and *S* is the surface area) for a given fish, h is a dummy variable expressing food type (1 for herbivores, and 0 for detritivores and carnivores), and *d* is a dummy variable also expressing food type (1 for detritivores, and 0 for herbivores and carnivores). The aspect ratio of each fish as well as *Q/B*s for the shellfishes were derived from Vibunpant *et al*. (2003).
4. Diet composition: the input on diet composition of each component was derived from relevant scientific reports on fish stomach contents in Bandon Bay and adjacent areas by DoF marine fishery scientists (Tables 4 and 5).
5. Inputs of non-fish and non-shellfish components: Biomass, *P/B* and *Q/B* of these components (benthos, zooplankton, phytoplankton and detritus) were derived from relevant literature (Supongpan *et al*. 2005a; 2005b; Sawusdee *et al*. 2009; Premcharoen 2012) and were assumed constant during the studied periods.

#### Model balancing

After input of all required parameters (biomass, *P/B* and *Q/B*, and diet composition data) into the model, a mass-balance was performed by modifying the entries until input and output were equal for each component (Webber *et al*. 2015). The criterion used for balancing the model was that the *EE* values for each component must be less than 1.0. If an *EE* value is more than 1, it indicates that predation on the component is greater than its production. Moreover, the gross efficiency (GE), i.e., food conversion efficiency, of each component in the system should range between 0.1 and 0.3 (Christensen *et al*. 2005). Thus, to meet the criteria for balancing the model, subtle adjustments were made for diet composition.

## RESULTS

Components (species/ species groups) in the models for 2007 and 2016 were similar except for stingrays, i.e., Family Dasyatidae, which were not recorded in the 2007 surveys. Some species were added to other component groups because their biomass was minimal during the two surveys (Table 1). Differences in biomass among the fishery resource components of Bandon Bay were observed after the ten-year interval. Most of the fish groups showed an increase in biomass, including the blue swimming crab *P. pelagicus*. A significant increase in biomass of blue swimming crab was observed despite high fishing pressure on this species, which was comparable between the two periods, and this may imply the success of the stocking programme (Table 2). On the other hand, three components showed significant decreases in biomass: other demersal fishes, cephalopods and Peaneid shrimps. The *P/B* values (estimated through *Z*-value) of most components in the 2016 model were a bit higher than 2007 models, except for *Lagocephalus* spp., pony fish, scads and *Upeneu*s spp. This is due to the smaller average size of the samples in 2016. Meanwhile *Q/B* values were set as constant in both models, i.e., assumes no difference in feeding rate of individual components. The trophic level (TL) of all components showed non-substantial changes, i.e., the difference in TL of each component between the two periods was less than 0.5, which implied their feeding plasticity. The TL of the blue swimming crab was 2.75 in 2007 and 2.54 in 2016.

**Figure 2:**
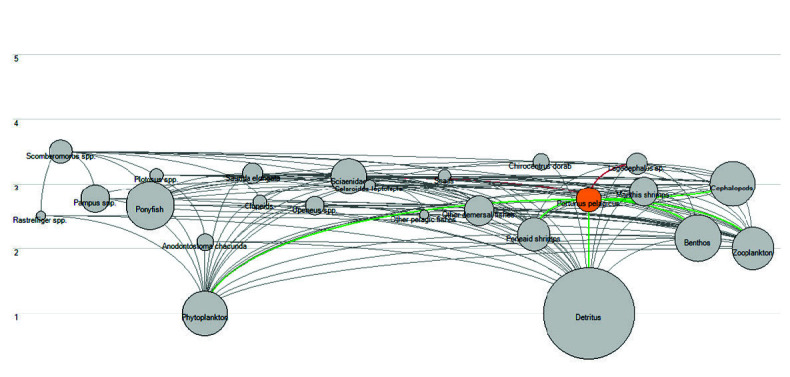

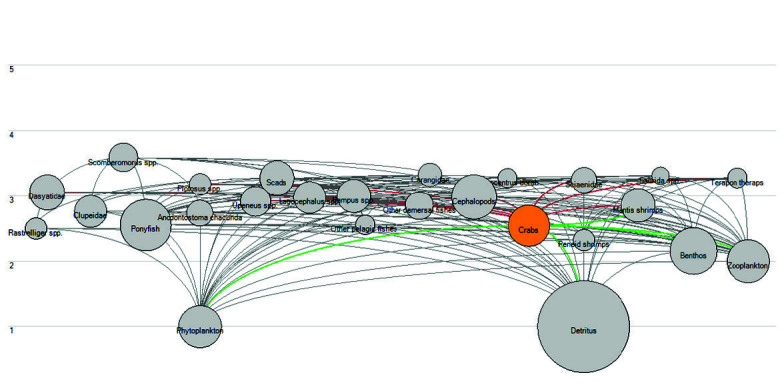
Flow-diagram of Bandon Bay ecosystem in two studied periods. (A) 2007 and (B) 2016.

The basic inputs and estimated parameters (*EE* and *GE*, as presented by *P/Q*) from the Ecopath model for Bandon Bay for 2007 and 2016 are presented in Table 2; the diet composition of each component is presented in Table 3. The *EE* values of all components were less than 1, and the *GE* values ranged between 0.1 and 0.3, meeting the requirements of a balanced model (Christensen *et al*. 2005) for both Ecopath models. The *EE* values for the shellfish components (> 0.5) were higher than the fish components (< 0.5), indicating that shellfish species were more heavily exploited than fishes in Bandon Bay. The blue swimming crab was among the components that were highly utilised both from within (through predation) and outside (through fisheries) the system, since its *EE* was close to 1.0. The *EE* values of the fish components were relatively low, indicating they were less predated on by the other components in the system. In terms of GE, i.e., food conversion efficiency, the value of 0.25 for the blue swimming crab indicated that consumption was four times higher than production. The balance network analysis (Fig. 2) shows the interactions and energy flows among components in the system. It is clear from this that the blue swimming crab mostly depended on the detrital-based food chain, i.e., the trophic interactions among recycling organic matter, detritus, predators on detritus (i.e., zoobenthos and zooplankton), and finally its predators.

Considering the system statistic estimates (see Table 6) for the Bandon Bay models, most of the ecological indices showed higher maturity and stability after 10 years of stocking blue swimming crabs. The throughput value of the 2007 phase (15071.19 t km^−2^ y^−1^) is a bit larger than the 2016 phase (11304.34 t km^−2^ y^−1^), which could be due to the fisheries in the Bandon Bay, which are mostly artisanal, except for the commercial blue swimming crab fishery. The Bandon Bay ecosystem became more mature from 2007 to 2016, as indicated by the total primary production per total respiration (TPP/TR), which was 2.06 in 2007 and 1.30 in 2016. The development of the Bandon ecosystem towards maturity during the 10 years of crab stocking also was reflected by higher values of system omnivory index (SOI), total number of pathways, and % ascendency in 2016 than in 2007. The higher total number of pathways and mean length of pathways in 2016 implied that the food web in the Bandon Bay ecosystem became more resistant to perturbation.

**Figure 3:**
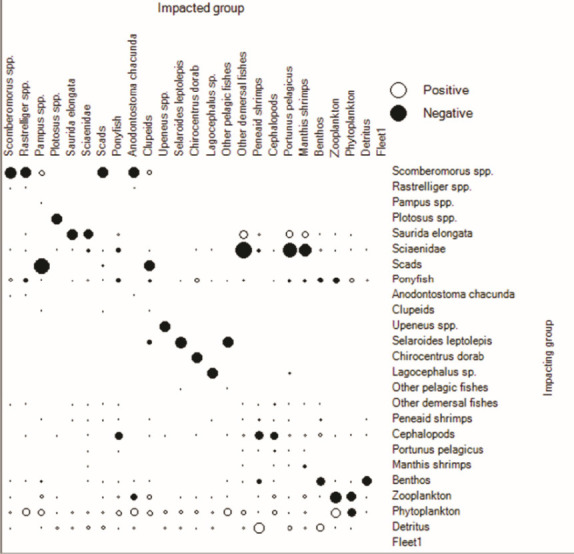

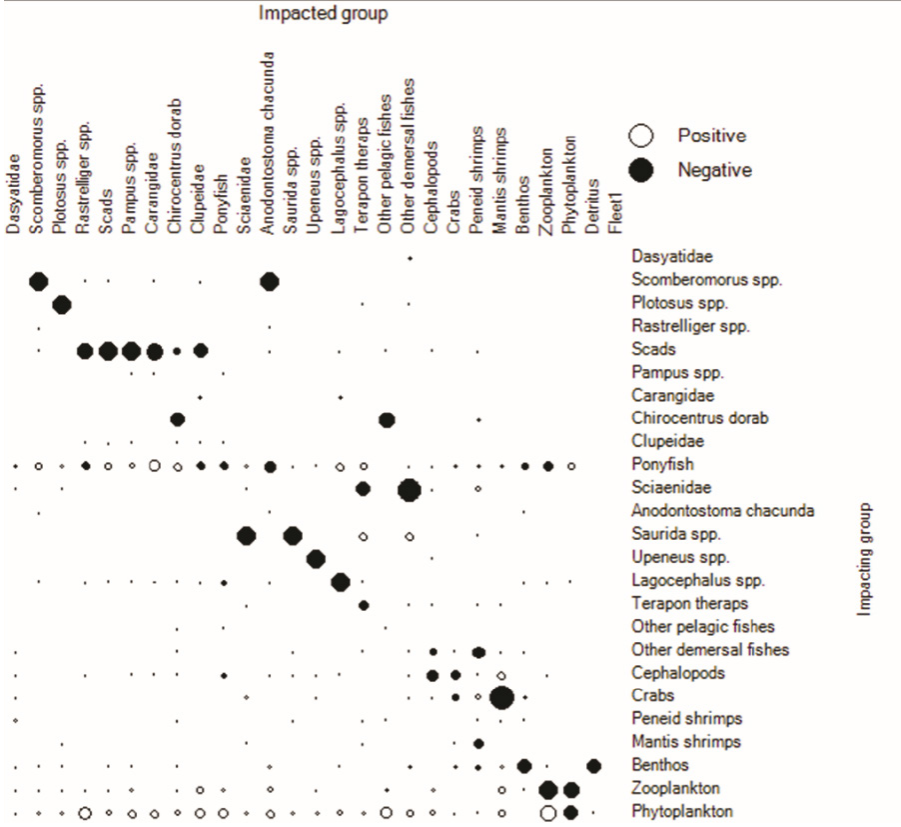
Mixed trophic impacts of Bandon Bay ecosystem in two studied periods. (A) 2007 and (B) 2016.

The mixed trophic impact (Fig. 3) describes the impact to all components in the system when the abundance of any impacting groups slightly increases, i.e., 10%. Increase of natural food sources (detritus, zooplankton, zoobenthos, phytoplankton and plants) showed positive impact on most of the remaining components, indicating bottom-up regulation in the Bandon Bay ecosystem. Increase in abundance of carnivorous fish (i.e., *TL* > 3), resulted in negative impact on most fish groups within this ecosystem as well as themselves, i.e., by cannibalism. The mixed trophic impacts (Fig. 3) clearly indicated that the increase in abundance of the blue swimming crab resulted in negative impact only to mantis shrimp by interspecific concentration, i.e., niche overlap.

## DISCUSSION

Applying the Ecopath model allows us to describe the trophic interactions and balance the biomass and annual production of key components in the Bandon Bay ecosystem before (2007) and after initiation of the crab bank programme (2016). The focus of the study was the blue swimming crab, which was continuously released into the studied area since 2010. Comparing the two Ecopath models showed differences in the food web structure and ecosystem properties in the Bandon Bay ecosystem that occurred during the 10 year interval. The major changes in the ecosystem properties of the bay were observed in the summary statistics attributes (Table 4), which showed improvement of ecosystem health. Although this improvement was certainly due to multiple causes, it may also be concluded that there was no negative effect to the ecosystem from the crab bank practice. It can be said that the Bandon Bay ecosystem became more mature, since TPP/TR in a mature ecosystem should be equal (Odum 1969); in this study the ratio decreased from 2.06 in 2007 to 1.30 in 2016. The connectivity index (CI) and (SOI) are correlated with system maturity because food chains generally change from linear to web-like as a system matures (Odum 1969; Khan *et al*. 2015). In this study, although CI did not change, SOI was higher in 2016, indicating the more web-like system. All flows and biomasses in the ECOPATH model can be shown in a single flow diagram as in Fig. 2, in which the size of the circles is proportional to biomass for each component and position on the *y*-axis represents trophic level. Also, according to Odum (1969), most components depended more on the detrital pathway, and this was apparent in 2016.

The *EE* values indicated that most components were substantially utilised, both from predation and exploitation in the system. It seems that the *EE* of most fish components in Bandon Bay were relatively low when compared to the whole GoT, for which values are always > 0.90 (Vibunpant *et al*. 2003; Supongpan *et al*. 2005a; 2005b). This could be explained by the bay *per se* acting as a nursery ground, and the fishing area and gears used are limited, mostly for artisinal fisheries (Jarernpornnipat *et al*. 2003; Sawusdee 2010). Moreover, the main fishery targets in the bay are shellfishes, i.e., squids, mantis shrimp, shrimps and blue swimming crab (Sawusdee 2010; Niumnuch & Purisumpun 2011), which also had higher *EE* than the fish components. The higher *EE* values for natural food sources (detritus, zooplankton, zoobenthos, phytoplankton and plant) indicated that they were nearly fully utilised by organisms in higher trophic levels (Khan *et al*. 2015); in particular, phytoplankton seems to be the base food source in the Bandon Bay ecosystem (Lursinsap 1982). The substantial increase in biomass of the blue swimming crab in 2016 likely led to a consequent increase in *EE* of the detritus and benthos, because of the crab’s bottom-feeding behaviour (Caddy & Defeo 2003).

Duldic *et al*. (1997) mentioned that coastal areas are usually comprised of low trophic level species with high ecological efficiency and productivity, which support the carnivores within or beyond the system. The majority of the biomass in 2007 and 2016 came from components with *TL* between 2 and 3. There was little variation in *TL* for these components in both periods, indicating that although they feed mainly on their preferred diet items, they have the capability for feeding plasticity (Panikkar & Khan 2008; Duan *et al*. 2009). Meanwhile, the decrease in TL of the blue swimming crab in 2016 may have been caused by intra-specific competition, whereby the increased abundance through stocking caused individuals to feed more often on detritus instead of the common prey, i.e. zoobenthos and zooplankton (Kunsook *et al*. 2014). The mixed trophic impact showed the characteristics of bottom-up control in the Bandon Bay ecosystem, in which changes in abundance of components with *TL* = 1 had positive impacts on most of the other components at higher trophic levels, and these impacts dominated ecosystem processes (Dyer & Letourneau 2003; Chassot *et al*. 2005). The possibility of a trophic cascade in Bandon Bay can also be considered. High fishing pressure on the shellfish components would result in a shift of diets of high-*TL* (i.e., > 3) components.

Jutagate and Sawusdee (2022) showed that the bottom-set gillnets and collapsible crab traps, the main fishing gears in blue swimming crab fisheries of Bandon Bay, are both focused exclusively on crabs, and that the crabs contributed over 50% of the index of relative importance of the catches. Considering the results of mixed trophic impacts, this implies that if there was excessive effort from both fishing gears, imbalance in the ecosystem would occur in the system. Some fishes such as ponyfish and fishes in Family Sciaenidae would be impacted by losing their preferred food source (i.e., blue swimming crab), and predate more on other invertebrates instead. Moreover, other species that were caught substatntially in either gear type, for example, horseshoe crab in gillnets and puffer fish and *Murex* snail in traps, would be reduced and consequently affect their prey and predator populations. Chassot *et al*. (2005) also stated that fishing generally affects species at higher trophic levels, which results in changes in their population dynamics and eventually alters the biomass of each component in the ecosystem.

## CONCLUSION

Two Ecopath models of Bandon Bay were constructed, for 2007 and 2016. The main objective was to understand the changes in the bay’s ecosystem after the inauguaration of the crab bank in 2007. Changes in most of the ecological indices revealed higher maturity and stability after 10 years of stocking by crab banks. Differences in abundance of each component between the two models were likely caused by fisheries. The bottom-up control of processes in the ecosystem of Bandon Bay was confirmed by the Ecopath model. Understanding the impacts of fishing activities on the ecosystem as well as examining likely top-down control processes (i.e., fishing control) in exploited ecosystem should receive focus for better resource and fisheries management of the productive Bandon Bay. Future work should also emphasise data quality and certainty of input parameters for better model performance.

## Figures and Tables

**Table 1:** Details of component, as group of species, in the Ecopath analysis of the Bandon Bay ecosystem. Each component includes the species that share the same niche.

Model	No.	Component	Including
2007	1	*Scomberomorus* spp.	*Scomberomorus commerson* and *S. tol*.
	2	*Pampus* spp.	*Pampus argenteus* and *Parastromateus niger*.
	3	Scads	*Alepes kleinii, Atule mate* and *Megalaspis cordyla*.
	4	Ponyfish	*Leiognathus elongatus, L. leuciscus and L. splendens, Secutor ruconius, S. insidiator* and *Pentaprion longimanus*.
	5	Clupeids	*Stolephorus indicus, Stolephorus sp., Engruaridae spp*.
	6	*Upeneus* spp.	*U. tragula* and *U. Sulphureus*.
	7	*Lagocephalus* spp.	*Lagocephalus lunaris* and *L. spadiceus*
	8	Other pelagic fishes	*Ilisha elongata* and all unidentified fishes in Family Mugilidae.
	9	Other demersal fishes	*Plectorhynchus pictus, Balistoides spp., Drepane punctata, Platychephalidae and Apogonidae*.
	10	Peneaid shrimps	*Metapenaeus lysianassa, M. palmensis, M. affinis* and *Penaeus merguiensis*.
	11	Cephalopods	*Photololigo duvaucelii, Sepiella innermis, Sepioteuthis lessoniana, Sepia pharaonis, Sepia recurvirostris*, and *Nipponololigo sumatrensis*.
	12	*Portunus pelagicus*	*Charybdis feriatus* and *Charybdis feriatus*.

2016	1	Dasyatidae	*Dasyatis* spp., *Himautura* spp. and *Maculabatis* spp.
	2	*Scomberomorus* spp.	*Scomberomorus commerson* and *Scomberomorus tol*.
	3	*Rastrelliger* spp.	*Rastrelliger brachysoma* and *Rastrelliger kanagurta*.
	4	*Pampus* spp.	*Pampus argenteus, P. chinensis* and *Parastromateus niger*.
	5	Scads	*Megalaspis cordyla, Atule mate, Alepes djeddaba, Alepes kleinii* and *Alepes melanoptera*.
	6	Carangidae	All unidentified fishes in Family Carangidae.
	7	Mugillidae	All unidentified fishes in Family Mugillidae.
	8	Ponyfish	*Leiognathus elongatus, L. leuciscus and L. splendens, Secutor ruconius, S. insidiator* and *Pentaprion longimanus*.
	9	Clupeids	*Stolephorus indicus, Stolephorus sp*. and *Engruaridae spp*.
	10	*Saurida* spp.	*Saurida elongata* and *S. isarankurai*.
	11	*Upeneus* spp.	*U. tragula* and *U. Sulphureus*.
	12	*Lagocephalus* sp.	*Lagocephalus lunaris* and *L. Spadiceus*.
	13	Other pelagic fishes	*Ilisha elongata* and all unidentified fishes in Family Mugilidae.
	14	Other demersal fishes	*Plectorhynchus pictus, Balistoides spp., Drepane punctata, Platychephalidae* and Apogonidae.
	15	Peneaid shrimps	*Metapenaeus lysianassa, M. palmensis, M. affinis* and *Penaeus merguiensis*.
	16	Cephalopods	*Photololigo duvaucelii, Sepiella innermis, Sepioteuthis lessoniana, Sepia pharaonis, Sepia recurvirostris*, and *Nipponololigo sumatrensis*.
	17	Crabs	*Portunus pelagicus, Charybdis feriatus* and *Charybdis feriatus*.

**Table 2:** Basic inputs (Biomass, P/B and Q/B) and estimated parameters (Trophic level, EE and P/Q) in the Ecopath model of the Bandon Bay ecosystem in 2007.

Group	Group name	Trophic level	Biomass (t/km^2^)	P/B (year ^−1^)	Q/B (year ^−1^)	EE	P/Q
1	*Scomberomorus* spp.	3.50	1.70	0.10	0.35	0.18	0.29
2	*Rastrelliger* spp.	2.50	0.20	2.56	12.00	0.06	0.21
3	*Pampus* spp.	2.77	3.39	0.88	4.40	0.05	0.20
4	*Plotosus* spp.	3.14	0.39	0.45	2.25	0.25	0.20
5	*Saurida elongata*	3.17	1.21	0.85	4.00	0.24	0.21
6	Sciaenidae	3.11	9.58	1.50	7.50	0.02	0.20
7	Scads	3.13	0.41	1.56	5.29	0.05	0.29
8	Ponyfish	2.67	48.38	3.50	14.00	0.35	0.25
9	*Anodontostoma chacunda*	2.10	0.67	1.81	10.75	0.02	0.17
10	Clupeids	2.72	0.43	2.70	12.00	0.36	0.23
11	*Upeneus* spp.	2.66	0.92	2.01	6.80	0.17	0.30
12	*Selaroides leptolepis*	2.99	0.30	2.22	11.80	0.27	0.19
13	*Chirocentrus dorab*	3.35	0.69	2.00	10.00	0.25	0.20
14	*Lagocephalus* sp.	3.32	1.35	3.00	12.00	0.20	0.25
15	Other pelagic fishes	2.52	0.21	4.00	16.00	0.21	0.25
16	Other demersal fishes	2.58	4.29	3.50	14.00	0.52	0.25
17	Peneaid shrimps	2.22	6.41	5.00	20.00	0.92	0.25
18	Cephalopods	3.00	31.95	1.30	5.20	0.52	0.25
19	*Portunus pelagicus*	2.75	2.25	2.50	10.00	0.78	0.25
20	*Manthis* shrimps	2.89	4.04	1.50	5.00	0.77	0.30
21	Benthos	2.16	33.00	5.00	25.00	0.94	0.20
22	Zooplankton	2.00	20.00	40.00	160.00	0.75	0.25
23	Phytoplankton	1.00	30.00	200.00		0.60	0.29
24	Detritus	1.00	10000.00	0.10		0.20	0.21

*Note:* P/B is production/biomass ratio, Q/B is consumption/biomass ratio, EE is ecotrophic efficiency, and P/Q is production/consumption ratio or gross efficiency (GE)

**Table 3:** Basic inputs (Biomass, P/B and Q/B) and estimated parameters (Trophic level, EE and P/Q) in the Ecopath model of the Bandon Bay ecosystem in 2016.

Group	Group name	Trophic level	Biomass (t/km^2^)	P/B (year ^−1^)	Q/B (year ^−1^)	EE	P/Q
1	Dasyatidae	3.04	8.25	0.50	2.50	0.00	0.20
2	*Scomberomorus* spp.	3.59	3.56	0.10	0.35	0.18	0.29
3	*Plotosus* spp.	3.18	1.33	0.55	2.25	0.25	0.24
4	*Rastrelliger* spp.	2.50	1.30	3.11	12.00	0.14	0.26
5	Scads	3.28	7.38	1.56	5.29	0.04	0.29
6	*Pampus* spp.	3.00	6.58	1.26	4.40	0.24	0.29
7	*Carangidae*	3.32	1.72	1.34	5.37	0.20	0.25
8	*Chirocentrus dorab*	3.28	1.00	2.00	10.00	0.45	0.20
9	Clupeidae	2.76	5.57	2.70	12.00	0.29	0.23
10	Ponyfish	2.56	58.67	3.50	14.00	0.62	0.25
11	Sciaenidae	3.25	2.35	1.50	7.50	0.06	0.20
12	*Anodontostoma chacunda*	2.73	2.52	1.81	10.75	0.01	0.17
13	*Saurida* spp.	3.31	1.14	2.27	4.00	0.09	0.57
14	*Upeneus* spp.	2.92	4.31	2.01	6.80	0.17	0.30
15	*Lagocephalus* spp.	2.98	5.36	3.00	12.00	0.23	0.25
16	*Terapon theraps*	3.28	1.10	2.15	10.00	0.67	0.22
17	Other pelagic fishes	2.56	0.97	4.00	16.00	0.38	0.25
18	Other demersal fishes	2.85	3.09	3.50	14.00	0.43	0.25
19	Cephalopods	2.98	26.59	1.30	5.20	0.61	0.25
20	Crabs	2.54	16.87	2.50	10.00	0.90	0.25
21	Peneid shrimps	2.32	1.36	5.00	20.00	0.96	0.25
22	Mantis shrimps	2.85	6.98	1.50	5.00	0.99	0.30
23	Benthos	2.16	33.00	5.00	25.00	0.94	0.20
24	Zooplankton	2.00	20.00	40.00	160.00	0.87	0.25
25	Phytoplankton	1.00	20.00	200.00		0.93	
26	Detritus	1.00	10000.00			0.49	

*Notes:* P/B is production/biomass ratio, Q/B is consumption/biomass ratio, EE is ecotrophic production/consumption ratio or gross efficiency (GE)

**Table 4:** Diet composition (vertical columns) of components for Ecopath analysis of Bandon Bay in 2007.

No.	Prey/Predators	1	2	3	4	5	6	7	8	9	10	11	12	13	14	15	16	17	18	19	20	21	22
1	*Scomberomorus* spp.	0.05																					
2	*Rastrelliger brachysoma*	0.05																					
3	*Pampus* spp.	0.05						0.05															
4	*Plotosus* spp.				0.05																		
5	*Saurida elongate*					0.05																	
6	Sciaenidae					0.05																	
7	Scads	0.05																					
8	Ponyfish	0.35		0.10	0.10	0.15	0.20	0.20					0.15	0.50	0.25	0.10			0.20				
9	*Anodontostoma chacunda*	0.05																					
10	Clupeids	0.05						0.10					0.05										
11	*Upeneus* spp.											0.05											
12	*Selaroides leptolepis*												0.05										
13	*Chirocentrus dorab*													0.05									
14	*Lagocephalus* sp.														0.05								
15	Other pelagic fishes												0.05										
16	Other demersal fishes	0.05			0.05	0.10	0.10																
17	Penaeid shrimps				0.05	0.10	0.10	0.05					0.05		0.10				0.10	0.10	0.05		
18	Cephalopods	0.10			0.10	0.05	0.05	0.05				0.05	0.05	0.10	0.10		0.05		0.05	0.10	0.05		
19	*Portunus pelagicus*						0.05								0.05								
20	Mantis shrimps				0.05		0.05														0.05		
21	Benthos				0.20	0.15	0.10	0.15	0.10		0.10	0.15	0.10		0.15		0.20	0.10		0.20	0.20	0.05	
22	Zooplankton	0.10	0.50	0.60	0.20	0.15	0.10	0.20	0.55	0.10	0.60	0.30	0.20	0.20	0.20	0.35	0.25	0.10	0.45	0.20	0.40	0.10	
23	Phytoplankton		0.40	0.20		0.10	0.10	0.05	0.25	0.70	0.15	0.25	0.15	0.05		0.45	0.25	0.10	0.10	0.10	0.05	0.20	1.00
24	Detritus	0.10	0.10	0.10	0.20	0.10	0.15	0.15	0.10	0.20	0.15	0.20	0.15	0.10	0.10	0.10	0.25	0.70	0.10	0.30	0.20	0.65	

	Total	1.00	1.00	1.00	1.00	1.00	1.00	1.00	1.00	1.00	1.00	1.00	1.00	1.00	1.00	1.00	1.00	1.00	1.00	1.00	1.00	1.00	1.00

*Note:* Number in the top row represents the group name of predator, as in column “Prey/Predators”

**Table 5:** Diet composition (vertical columns) of components for Ecopath analysis of Bandon Bay in 2016.

No.	Prey/Predators	1	2	3	4	5	6	7	8	9	10	11	12	13	14	15	16	17	18	19	20	21	22	23	24
1	Dasyatidae																								
2	*Scomberomorus* spp.		0.05																						
3	*Plotosus* spp.			0.05																					
4	*Rastrelliger* spp.		0.05			0.01																			
5	Scads		0.05			0.01																			
6	*Pampus* spp.		0.05			0.05																			
7	Carangidae		0.05			0.01																			
8	*Chirocentrus dorap*					0.01			0.05																
9	Clupeidae		0.05			0.10		0.05																	
10	Ponyfish		0.45	0.25		0.45	0.45	0.60	0.50	0.10		0.20		0.15		0.50	0.35	0.20	0.15	0.20					
11	Sciaenidae													0.05											
12	*Anodontostoma chacunda*		0.05																						
13	*Saurida* spp.													0.05											
14	*Upeneus* spp.														0.05										
15	*Lagocephalus* spp.							0.05								0.05									
16	*Terapon theraps*			0.05								0.05					0.05								
17	Other pelagic fishes		0.05			0.01			0.10																
18	Other demersal fishes	0.10		0.05								0.10		0.15											
19	Cephalopods	0.05	0.05	0.05		0.05		0.05				0.10		0.10	0.10		0.10		0.10	0.05					
20	Crabs	0.10		0.05								0.10			0.10		0.10		0.10	0.10	0.05		0.10		
21	Penaeid shrimps								0.10			0.05		0.05			0.05		0.05				0.05		
22	Mantis shrimps	0.05		0.05										0.05			0.05				0.05				
23	Benthos	0.35		0.05						0.05	0.05	0.10	0.20	0.10	0.15		0.05			0.10	0.15	0.15	0.20	0.05	
24	Zooplankton	0.10		0.15	0.50	0.10	0.30	0.10	0.10	0.55	0.50	0.10	0.50	0.10	0.30	0.10	0.05	0.25	0.20	0.30	0.20	0.15	0.40	0.10	
25	Phytoplankton				0.40	0.05	0.15	0.15	0.05	0.20	0.30	0.10	0.20	0.10	0.10	0.15	0.10	0.45	0.20	0.10	0.10	0.10	0.05	0.20	1.00
26	Detritus	0.25	0.10	0.25	0.10	0.15	0.10		0.10	0.10	0.15	0.10	0.10	0.10	0.20	0.20	0.10	0.10	0.20	0.15	0.45	0.60	0.20	0.65	

	Total	1.00	1.00	1.00	1.00	1.00	1.00	1.00	1.00	1.00	1.00	1.00	1.00	1.00	1.00	1.00	1.00	1.00	1.00	1.00	1.00	1.00	1.00	1.00	1.00

*Note:* Number in the top row represents the group name of predator, as in column “Prey/Predators”

**Table 6:** System statistics estimated for pre-stock (2007) and post-stock (2016) phases for comparing the status of Bandon Bay ecosystem.

Parameter	2007	2016	% difference
Total system throughput (TST) *	1,5071.19	1,1304.34	0.91
Sum of all flows into detritus *	3,841.63	1,757.264	−0.54
Total biomass/TST	0.01	0.02	1.00
Total primary production/total respiration	2.06	1.30	−0.37
Connectance index	0.25	0.25	0.00
System omnivory index	0.28	0.32	0.14
Total number of pathways	113	140	0.24
Mean length of pathways	3.65	4.16	−0.14
Ascendency (%)	32.8	28.2	0.07
Overhead (%)	67.1	71.7	0.20

*Note:*

* = unit: t/km^2^/yr
